# Book Reviews

**Published:** 1891-08

**Authors:** 


### Book Reviews.

A PRACTICAL TREATISE ON DISEASES OF THE SKIN. By Henry G. Piffard, A. M., M. D., Clinical Professor of Dermatology, University of the City of New York. Assisted by Robert M. Fuller, M. D. New York. D. Appleton & Co.

An eminent dermatologist said of the numerous pictures of skin diseases, that you might place one of them before a number of dermatologists, and each one, not seeing the name beneath, would call the representation a different disease. The plates and cuts in the work of Dr. Piffard are better than some which have been presented, yet we find the same objection as above mentioned.

One notable departure in the work is the failure to attempt a set classification of the various skin eruptions. This, alone, is an advantage in favor of the work, for nothing is more difficult and confusing than the effort to keep up with the classifications given by the various authors.

As we would expect from one of Dr. Piffard's ability, the text is well written, practical and will prove a useful guide to physicians who consult its contents.

He closes the work with an exposition of his own views regarding psorosperms, to which various diseases of the skin have, within the past few years, been attributed. M. B. H.

---

DR. JOHN B. HAMILTON has resigned the position of Surgeon General U. S. Marine Hospital Service, to accept the Professorship of Surgery in the Rush Medical College, Chicago. Dr. Walter Wayman, for many years Dr. Hamilton's chief assistant in the Marine Hospital Service, succeeds him as Surgeon General.

